# Risk Factors for Survival in Patients With Medulloblastoma: A Systematic Review and Meta-Analysis

**DOI:** 10.3389/fonc.2022.827054

**Published:** 2022-03-03

**Authors:** Yu Liu, Bo Xiao, Sen Li, Jiangang Liu

**Affiliations:** Department of Neurosurgery, Shanghai Children’s Hospital, Shanghai Jiao Tong University, Shanghai, China

**Keywords:** medulloblastoma, survival, prognosis, histology, molecular typing, radiotherapy, meta-analysis

## Abstract

**Background:**

Conventional parameters show limited and unreliable correlations with medulloblastoma prognosis.

**Aim:**

To evaluate the factors influencing overall survival (OS), event-free survival (EFS), and progression-free survival (PFS) in patients with medulloblastoma.

**Methods:**

PubMed, EMBASE, the Cochrane Library, and Web of Science were searched for studies published up to May 2021. The associations between various clinical and treatment factors and survival parameters were assessed.

**Results:**

Twenty-nine studies (8455 patients) were included. Desmoplastic medulloblastoma (HR=0.41, 95%CI: 0.31-0.56), M0 disease (HR=2.07, 95%CI: 1.48-2.89), WNT, SSH, group 4 (all P<0.05 vs. group 3), GTR vs. STR (HR=1.37, 95%CI: 1.04-1.08), radiotherapy (HR=0.45, 95%CI: 0.20-0.80), craniospinal irradiation (HR=0.49, 95%CI: 0.38-0.64), and high 5hmC levels (HR=2.90, 95%CI: 1.85-4.55) were associated with a better OS. WNT, SSH, group 4 (all P<0.05 vs. group 3), residual tumor ≤1.5 cm^2^ (HR=2.08, 95%CI: 1.18-3.68), GTR vs. STR (HR=1.31, 95%CI: 1.03-1.68), craniospinal irradiation (HR=0.46, 95%CI: 0.37-0.57), high 5hmC levels (HR=3.10, 95%CI: 2.01-4.76), and <49 days between resection and radiotherapy (HR=2.54, 95%CI: 1.48-4.37) were associated with better PFS. Classic vs. desmoplastic medulloblastoma (HR=1.81, 95%CI: 1.04-3.16), SSH, WNT (both P<0.05 vs, non-SSH/non-WNT), GTR vs. STR (HR=2.01, 95%CI: 1.42-2.85), and radiotherapy (HR=0.31, 95%CI: 0.15-0.64) were associated with a better EFS.

**Conclusion:**

Histology, molecular subgroup, GTR, and radiotherapy are significantly associated with survival parameters in patients with medulloblastoma. Nevertheless, high-quality prospective cohort studies are necessary to improve the conclusions.

## Introduction

Medulloblastoma (MB) is a malignant embryonal tumor of the cerebellum and represents over 20% of all central nervous system (CNS) neoplasms in children (1–3). The incidence of MB is 3.8-6.9 per million children in North America and Europe (2, 4–6). MB occurs most often in children aged 1-10 years, with peaks in children aged 3-4 and 8-10 years (2, 3). The treatment of MB is multidisciplinary and includes surgery, radiotherapy, and chemotherapy (7). Unfortunately, despite the best care, the 10-year mortality rate of MB is 34.6% in children (8). Therefore, assessing the prognostic and therapeutic actors is indispensable for managing patients with MB.

MB patients are stratified into standard- and high-risk groups according to clinical presentation, amount of residual disease after definitive surgery, tumor histopathology grouping, and biological or molecular tumor cell characteristics (2, 3, 9). Standard-risk patients ≥3 years old have a 5-year overall survival (OS) of >70% on current treatment protocols, including surgery, craniospinal irradiation, and chemotherapy (2, 3). High-risk patients ≥3 years old have a 5-year event-free survival (EFS) of about 70% for patients with metastatic MB receiving intensified chemotherapy regimens (myeloablative schedules with hematopoietic support of peripheral harvested stem cells), nonconventional radiation therapy schedules, and concurrent radiation and radiosensitizers schedules (2, 3). Children ≤3 years old have a 5-year progression-free survival (PFS) of 30%-90%, depending on tumor histology in this age group (2, 3). The classic and large cell/anaplastic subtypes are associated with a poor prognosis, while the desmoplastic/nodular and MB with extensive nodularity subtypes are associated with a better prognosis (2, 3). The wingless/integrated (WNT) subtype is associated with an excellent prognosis, the sonic hedgehog (SHH) and group 4 subtypes are associated with an intermediate prognosis, while the group 3 subtype is associated with a poor prognosis (2, 3).

Nonetheless, these parameters show limited and unreliable correlations with the prognosis of MB (10–12). Systematic reviews quantitatively assessing the potential risk factors have been published in 2010 and 2016 (13, 14), but several papers have been published since (12, 15–17), providing recent assessments of traditional risk factors and new ones.

Therefore, this meta-analysis aimed to evaluate the factors influencing OS, EFS, PFS, and relapse-free survival (RFS) in patients with MB. The results could help a better stratification of the patients and eventually improve management.

## Methods

### Literature Search

This systematic review and meta-analysis was reported according to the Preferred Reporting Items for Systematic Reviews and Meta-Analyses (PRISMA) guidelines (18, 19). PubMed, EMBASE, the Cochrane Library, and Web of Science were systematically searched for studies published up to May 2021. For the search, we used the Mesh term of ‘Medulloblastoma’ (for it was the disease of interest), ‘Prognosis’ (for it was the outcome of interest), and ‘Prospective Studies’, and ‘cohort’ (for they were the desired types of studies), as well as relevant key words. The search was performed independently and in parallel by two investigators (** and **). This included the analysis of titles/abstracts followed by the full texts. Disagreements were solved by a third investigator (**).

### Eligibility

The inclusion criteria were 1) cohort or cross-sectional studies investigating risk factors for mortality, 2) medulloblastoma confirmed pathologically, 3) reported outcome measures with hazard ratios (HRs) with 95% confidence intervals (CIs), and 4) published in English or Chinese. The exclusion criteria were 1) letters, review articles, meta-analysis, case-control, case reports, or animal studies, 2) missing primary data, 3) unpublished data, or 4) full text unavailable.

### Data Extraction and Quality Assessment

Data including authors’ names, publication year, study design, sample size, age at diagnosis, population, the extent of resection, location, radiotherapy, chemotherapy source of subjects, histological type, molecular subtype, and male percentage were extracted by two investigators (** and **). Any discrepancies in the characteristics of the studies and data extracted for meta-analysis between the two investigators were resolved by a third investigator (**) after reviewing the disputed data against the original publication. The study outcome was the association between factors and overall survival (OS), event-free survival (EFS), progression-free survival (PFS), and recurrence-free survival (RFS).

The methodological quality of the cohort studies was evaluated using the Newcastle-Ottawa Scale (NOS) (20), with a maximum of 9 stars, representing the least risk of bias. The quality assessment was performed in duplicate by two investigators separately (** and **).

### Statistical Analysis

Crude HRs with their 95% CIs were estimated and used to assess the strength of association between factors and OS, EFS, PFS, and RFS. The pooled HRs were calculated for demographic characteristics, clinical characteristics, and treatment history. The pooled HRs were determined using the Z-test (P ≤ 0.05). Cochran’s Q statistic (P<0.10 indicated evidence of heterogeneity) was used to assess the heterogeneity among studies (21). When significant heterogeneity (P<0.10) was achieved, the random-effects model was used to combine the effect sizes of the included studies; otherwise, the fixed-effects model was adopted (22). In addition, sensitivity analyses were performed to identify individual study effects on the pooled results and test the reliability of results. All analyses were performed using STATA SE 14.0 (StataCorp, College Station, Texas, USA).

## Results

### Selection of the Studies

**Figure 1:**
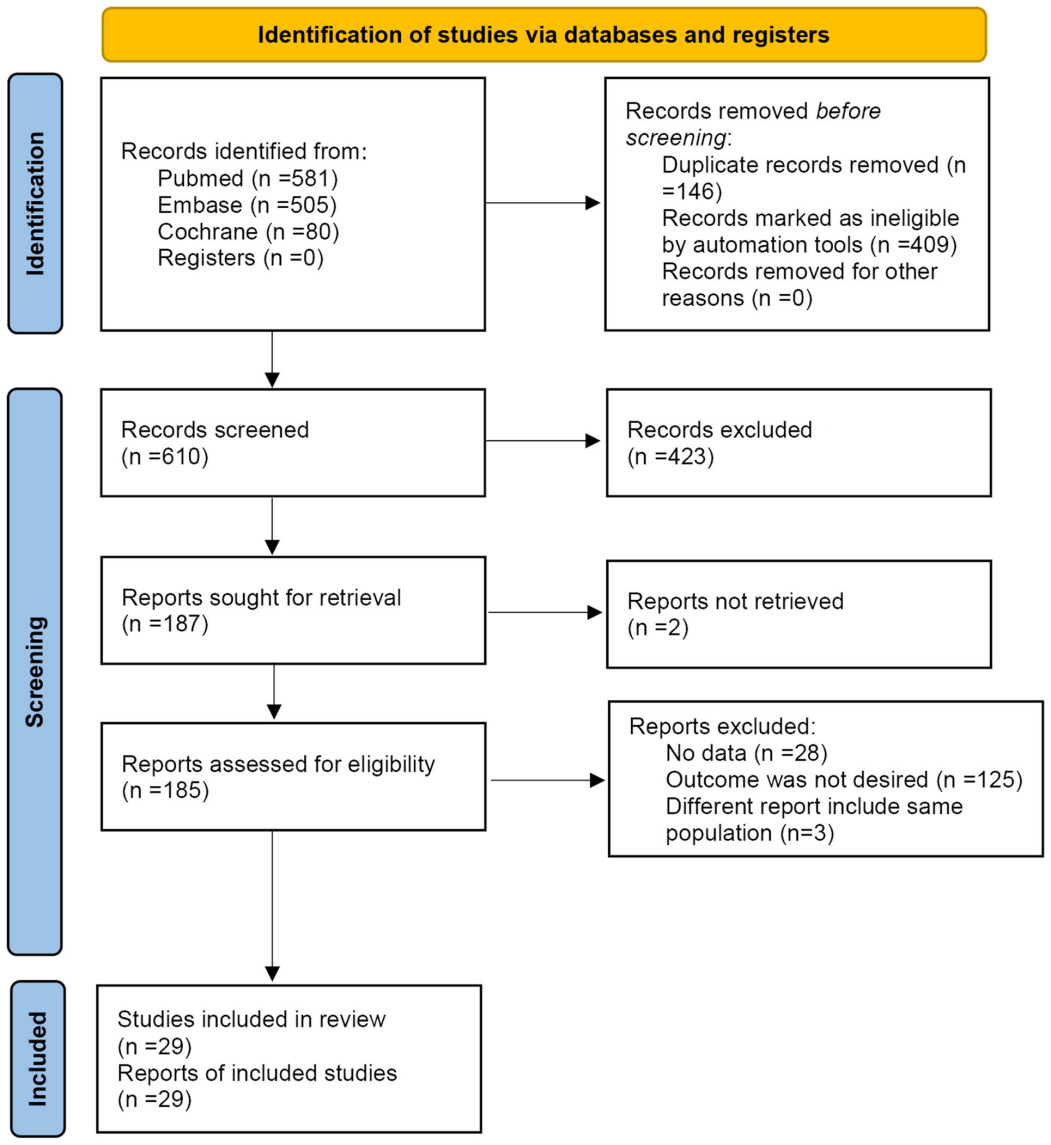
Flowchart of the search process.

**Figure 1** presents the study selection process. The initial search resulted in 1166 records, but 555 were removed before the screening. Then, 610 records were screened, and 423 were excluded. Among the 187 reports sought for retrieval, two could not be retrieved. Among the 185 reports assessed for eligibility, 156 were excluded (no data, n=28; outcomes were not desired, i.e., did not report OS, PFS, or EFS, n=125; different reports of the same populations, n=3).

### Characteristics of the Included Studies

**Supplementary Table S1** presents the characteristics of the included studies. The 29 studies included 8455 patients. There were 24 cohort studies, four database studies, and one international meta-analysis (although that study termed itself a meta-analysis (14), it was, in fact, a kind of retrospective cohort study based on the pooled data from five trials, not an actual meta-analysis). Thirteen studies included adults. Follow-up ranged from 1.8 to 9.3 years. On the NOS, nine studies scored 6 stars, two scored 7 stars, 15 scored 8 stars, and two scored 9 stars (**Table 1**). RFS could not be analyzed because of a lack of data in the included studies.

**Table 1:** Quality assessment of the included studies.

Study	Representativeness of the Exposed Cohort	Selection of the Non-Exposed Cohort	Ascertainment of Exposure	Demonstration that the Outcome of Interest was not Present at the Start of the Study	Comparability of Cohorts Based on the Design or Analysis	Assessment of Outcome	Was Follow-Up long Enough for the Outcomes to Occur	Adequacy of Follow Up of Cohorts	Total
Arroyo et al. (23)	/	✩	✩	✩	✩✩	✩	✩	✩	8
Back et al. (24)	/	✩	✩	✩	✩✩	✩	✩	✩	8
Bleil et al. (25)	/	✩	✩	✩	✩✩	✩	✩	✩	8
Brasme et al. (26)	✩	✩	✩	✩	✩✩	✩	✩	✩	9
Chin et al. (27)	/	✩	✩	✩	✩✩	✩	✩	✩	8
Dietzsch et al. (28)	/	✩	✩	✩	✩✩	✩	✩	✩	8
Eaton et al. (29)	/	✩	✩	✩	✩✩	✩	✩	✩	8
Hill et al. (30)	/	✩	✩	✩	✩✩	✩	✩	✩	8
Lai (31)	✩	✩	✩	✩	✩✩	✩	✩	✩	9
Li et al. (32)	/	✩	✩	✩	✩✩	✩	/	/	6
Li et al. (33)	/	✩	✩	✩	✩✩	✩	/	/	6
Massimino et al. (34)	/	✩	✩	✩	✩✩	✩	✩	✩	8
Nalita et al. (35)	/	✩	✩	✩	✩✩	✩	✩	✩	8
Ozer et al. (36)	/	✩	✩	✩	✩✩	✩	/	/	6
Padovani et al. (37)	/	✩	✩	✩	✩✩	✩	✩	✩	8
Pietsch et al. (11)	/	✩	✩	✩	✩✩	✩	✩	✩	8
Qin et al. (38)	✩	✩	✩	✩	✩✩	✩	/	/	7
Riffaud et al. (39)	/	✩	✩	✩	✩✩	✩	✩	✩	8
Rutkowski et al. (14)	/	✩	✩	✩	✩✩	✩	✩	✩	8
Schwalbe et al. (40)	/	✩	✩	✩	✩✩	✩	✩	✩	8
Soon et al. (16)	✩	✩	✩	✩	✩✩	✩	/	/	7
Thompson et al, (41)	/	✩	✩	✩	✩✩	✩	/	/	6
Wang et al. (12)	/	✩	✩	✩	✩✩	✩	/	/	6
Weil et al. (42)	/	✩	✩	✩	✩✩	✩	/	/	6
Yehia et al. (43)	/	✩	✩	✩	✩✩	✩	/	/	6
Yu and Li (44)	/	✩	✩	✩	✩✩	✩	/	/	6
Zhao et al. (45)	/	✩	✩	✩	✩✩	✩	✩	✩	8
Zhao et al. (17)	/	✩	✩	✩	✩✩	✩	/	/	6

Representativeness of the exposed cohort: one star if truly/somewhat representative of the average exposure in the community. Selection of the non-exposed cohort: one star if drawn from the same community as the exposed cohort. Ascertainment of exposure: one star if secure records or structured interview. Demonstration that the outcome of interest was not present at the start of the study: one star if yes. Comparability of cohorts based on the design or analysis: one star if the study controls for the most important factor; one additional star if the study controls for additional factors. Assessment of outcome: one star if the outcome is independently and blindly assessed or if record linkage. Was follow-up long enough for the outcomes to occur: one star if yes. Adequacy of follow up of cohorts: one star if all subjects are accounted for or if the subjects lost to follow-up will not bias the results. The maximum number of stars is 9.

✩: Included studies quality assessment. One star for matching one item. ✩✩: Included studies quality assessment. Two stars for matching two items. /: Not applicable.

### Overall Survival

**Figure 2:**
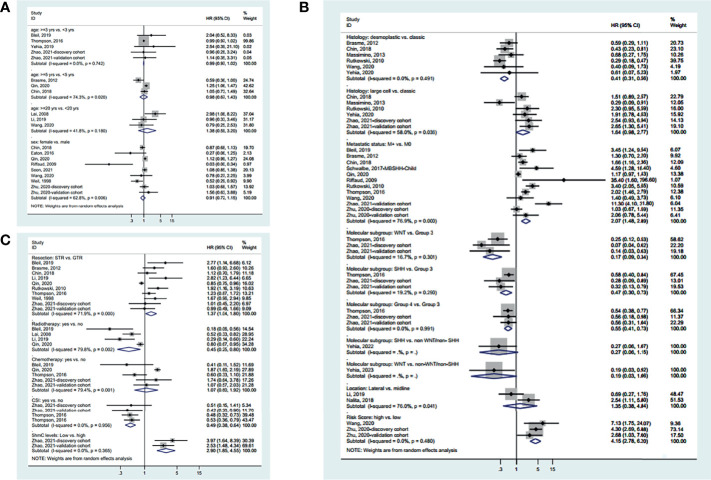
Forest plots of the prognostic factors and overall survival (OS). **(A)** Demographic characteristics. **(B)** Clinical characteristics. **(C)** Treatment history.

Different age cutoff points were used in the various studies. Still, age was not associated with OS irrespective of the cutoff point (3 years: HR=0.99, 95%CI: 0.97-1.02, I^2^ = 0.0%, P_heterogeneity_=0.742; 5 years: HR=0.98, 95%CI: 0.67-1.43, I^2^ = 74.3%, P_heterogeneity_=0.020; 20 years: HR=1.38, 95%CI: 0.59-3.20, I^2^ = 41.8%, P_heterogeneity_=0.180) (**Figure 2A**). Sex was not associated with OS (HR=0.91, 95%CI: 0.72-1.15, I^2^ = 62.8%, P_heterogeneity_=0.006) (**Figure 2A**).

Regarding the characteristics of the disease (**Figure 2B**), desmoplastic MB appears to have a better OS than classic MB (HR=0.41, 95%CI: 0.31-0.56, I^2^ = 0.0%, P_heterogeneity_=0.491), while there was no significant difference between large cell and classic MB (HR=0.98, 95%CI: 0.67-1.43, I^2^ = 74.3%, P_heterogeneity_=0.020). Metastatic disease is associated with a worse OS (HR=2.07, 95%CI: 1.48-2.89, I 2 = 76.9%, P_heterogeneity_<0.001). Regarding the molecular subtypes, WNT had a better OS than group 3 (HR=0.17, 95%CI: 0.09-0.34, I^2^ = 16.7%, P_heterogeneity_=0.301), SHH had a better OS than group 3 (HR=0.47, 95%CI: 0.30-0.73, I^2^ = 19.2%, P_heterogeneity_=0.290), and group 4 had a better OS than group 3 (HR=0.55, 95%CI: 0.41-0.73, I^2^ = 0.0%, P_heterogeneity_=0.991), while there was no difference in OS between SHH and non-WNT/non-SHH (HR=0.27, 95%CI: 0.06-1.15) and between WNT and non-WNT/non-SHH (HR=0.19, 95%CI: 0.03-1.06). There was no difference in OS between lateral and midline MB (HR=1.35, 95%CI: 0.38-4.84, I^2^ = 76.0%, P_heterogeneity_=0.041). Standard-risk MB had a better OS than high-risk MB (HR=4.15, 95%CI: 2.78-6.20, I^2^ = 0.0%, P_heterogeneity_=0.480).

Regarding the treatments (**Figure 2C**), gross tumor resection (GTR) achieves a better OS than subtotal resection (STR) (HR=1.37, 95%CI: 1.04-1.08, I^2^ = 71.9%, P_heterogeneity_<0.001). Radiotherapy (HR=0.45, 95%CI: 0.20-0.80, I^2^ = 79.8%, P_heterogeneity_=0.002) and craniospinal irradiation (HR=0.49, 95%CI: 0.38-0.64, I^2^ = 0.0%, P_heterogeneity_=0.956) improve OS. Chemotherapy did not influence OS (HR=1.07, 95%CI: 0.60-1.92, I^2^ = 79.4%, P_heterogeneity_=0.001). High 5hmC levels are associated with a better OS (HR=2.90, 95%CI: 1.85-4.55, I^2^ = 0.0%, P_heterogeneity_=0.365). The summarized results are presented in **Supplementary Figure S1A**.

### Progression-Free Survival

**Figure 3:**
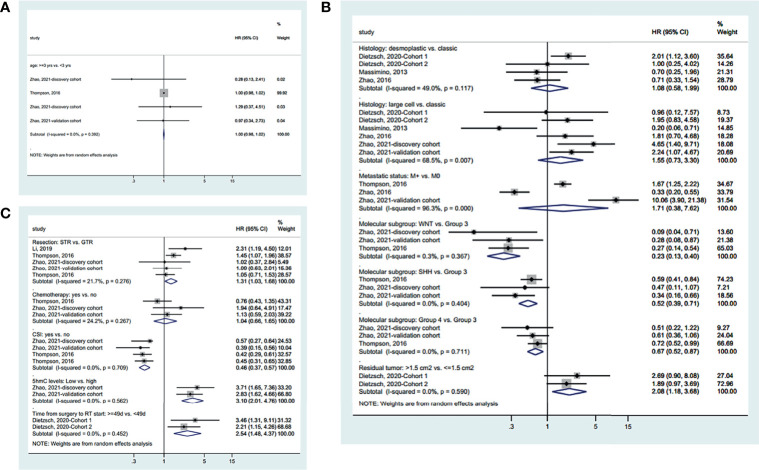
Forest plots of the prognostic factors and progression-free survival (PFS). **(A)** Demographic characteristics. **(B)** Clinical characteristics. **(C)** Treatment history.

Age was not associated with PFS based on a cutoff point of 3 years (HR=1.00, 95%CI: 0.98-1.02, I^2^ = 0.0%, P_heterogeneity_=0.392) (**Figure 3A**). There were no differences in PFS between desmoplastic and classic MC (HR=1.08, 95%CI: 0.58-1.99, I^2^ = 49.0%, P_heterogeneity_=0.117), between large cell and classic MB (HR=1.55, 95%CI: 0.73-3.30, I^2^ = 68.5%, P_heterogeneity_=0.007), and between metastatic and non-metastatic disease (HR=1.71, 95%CI: 0.38-7.62, I^2^ = 96.3%, P_heterogeneity_<0.001). Compared with group 3 MB, WNT (HR=0.23, 95%CI: 0.13-0.40, I^2^ = 0.3%, P_heterogeneity_=0.367), SHH (HR=0.52, 95%CI: 0.39-0.71, I^2^ = 0.0%, P_heterogeneity_=0.404), and group 4 (HR=0.67, 95%CI: 0.52-0.87, I^2^ = 0.0%, P_heterogeneity_=0.711) MB had a better PFS. Residual tumor ≤1.5 cm^2^ had a better PFS that >1.5 cm^2^ (HR=2.08, 95%CI: 1.18-3.68, I^2^ = 0.0%, P_heterogeneity_=0.290) (**Figure 3B**).

Regarding the treatments (**Figure 3C**), GTR improved PFS compared with STR (HR=1.31, 95%CI: 1.03-1.68, I^2^ = 21.7%, P_heterogeneity_=0.0.276). Chemotherapy did not influence PFS (HR=1.04, 95%CI: 0.66-1.65, I^2^ = 24.2%, P_heterogeneity_=0.267). CSI improved PFS (HR=0.46, 95%CI: 0.37-0.57, I^2^ = 0.0%, P_heterogeneity_=0.709). High 5hmC levels (HR=3.10, 95%CI: 2.01-4.76, I^2^ = 0.0%, P_heterogeneity_=0.562) and less than 49 days between surgery and radiotherapy (HR=2.54, 95%CI: 1.48-4.37, I^2^ = 0.0%, P_heterogeneity_=0.452) were associated with a better PFS. The summarized results are presented in **Supplementary Figure S1B**.

### Event-Free Survival

**Figure 4:**
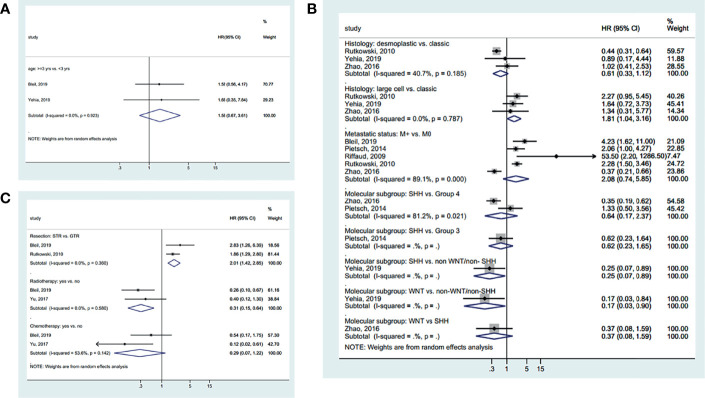
Forest plots of the prognostic factors and event-free survival (EFS). **(A)** Demographic characteristics. **(B)** Clinical characteristics. **(C)** Treatment history.

Age was not associated with EFS based on a cutoff point of 3 years (HR=1.56, 95%CI: 0.67-3.61, I^2^ = 0.0%, P_heterogeneity_=0.903) (**Figure 4A**). Classic MB had a better EFS than desmoplastic MB (HR=1.81, 95%CI: 1.04-3.16, I^2^ = 0.0%, P_heterogeneity_=0.787) (**Figure 4B**). SHH (HR=0.25, 95%CI: 0.07-0.89) and WNT (HR=0.17, 95%CI: 0.03-0.90) MB had a better EFS than non-WNT/non-SHH MB. There were no significant differences in the other tumor characteristics (**Figure 4B**). GTR (vs. STR) (HR=2.01, 95%CI: 1.42-2.85, I^2^ = 0.0%, P_heterogeneity_=0.360) and radiotherapy (HR=0.31, 95%CI: 0.15-0.64, I^2^ = 0.0%, P_heterogeneity_=0.580) improved EFS, while chemotherapy had no significant effect on EFS (HR=0.29, 95%CI: 0.07-1.22, I^2^ = 53.6%, P_heterogeneity_=0.142) (**Figure 4C**). The summarized results are presented in **Supplementary Figure S1C**.

### Publication Bias

**Supplementary Figure S2A** suggests no publication bias regarding the OS of metastatic vs. non-metastatic disease, but the funnel plot of STR vs. GTR shows an asymmetric distribution, suggesting publication bias (**Supplementary Figure S2B**).

### Sensitivity Analyses

The sensitivity analyses suggest that the results are robust, without any single study influencing the results by itself (**Supplementary Figure S3**).

## Discussion

The conventional prognostic parameters [amount of residual disease after definitive surgery, tumor histopathology grouping, and biological or molecular tumor cell characteristics (2, 3, 9)] show limited and unreliable correlations with the prognosis of MB. Therefore, this meta-analysis aimed to evaluate the factors influencing the survival parameters in patients with medulloblastoma. The results suggest that histological characteristics, molecular subgroups, GTR, and radiotherapy are significantly associated with survival parameters in patients with medulloblastoma.

Three previous meta-analyses on the potential risk factors for survival to MB have been published in 2010, 2016, and 2019 (13, 14, 46). Kocabaya et al. (13) (907 patients) reported that incomplete resection, brainstem infiltration, no chemotherapy, and no radiotherapy were significantly associated with poor OS, while metastatic disease and histologic subtypes were not associated. Rutkowski et al. (14) (270 children) reported that histologic subtype, incomplete resection, metastatic MB, and national groups were significantly associated with EFS and OS. The meta-analysis by Sharma et al. (46) focused on Groups 3 and 4; it highlighted molecular profiling differences in those patients and suggested refinements in the molecular classification. The present meta-analysis included 8455 patients and studies performed in the era of molecular typing.

In the present meta-analysis, age was not associated with OS, PFS, or EFS, irrespective of using the traditional cutoff point of 3 years or other cutoff points such as 5 or 20 years. Nevertheless, age is considered a parameter in the new molecular grouping, especially in Wnt and SHH types, which are seen in older children, adolescents, and adults. Still, a conventional prognostic factor like age might now be overwhelmed by the molecular grouping of MB. Although the risk level was associated with OS in the present meta-analysis, these results conflict with the general principles of MB prognosis that consider the combination of age and risk level for prognosis and guiding management (2, 3). Younger children generally have a poor prognosis because of undeveloped CNS and aggressive disease (2, 3), but the present meta-analysis suggests that age is not representative of disease aggressiveness.

On the other hand, the present meta-analysis suggests that the histologic subtypes are associated with survival, with the classic subtype having the poorest OS, the large cell subtype having a poor EFS, and the desmoplastic subtype having a good OS. These results are supported by the general view that the classic and large cell/anaplastic subtypes are associated with a poor prognosis and that the desmoplastic/nodular and MB with extensive nodularity subtypes are associated with a better prognosis (2, 3, 14, 39, 47). Regarding molecular subtypes, the present meta-analysis also suggests that group 3 MB has the worst prognosis among all MBs, as supported by the literature (2, 3, 7, 11, 28, 30, 32, 33, 40, 41, 43, 45, 47).

Regarding the treatments, the present meta-analysis suggests that GTR and radiotherapy are the major treatment parameters improving OS, PFS, and EFS, while chemotherapy had no impact on survival. These results contradict the global view and guidelines stating that chemotherapy is a standard treatment for MB (2, 3, 48, 49). Nevertheless, a Cochrane meta-analysis suggests that adjuvant chemotherapy and postoperative radiation therapy may not improve overall survival in patients < 21 years old with medulloblastoma (50). Furthermore, a study showed no significant difference in progression-free survival or overall survival associated with or without cisplatin maintenance chemotherapy following radiation therapy in patients with high-risk primary neuroectodermal tumors (51). On the other hand, the role of radiotherapy in managing MB is well supported by the literature (2, 3, 33, 45, 48, 49, 52). Furthermore, delayed radiotherapy has been associated with decreased OS (53), supporting the present meta-analysis suggesting a worse PFS for ≥49 days between surgery and radiotherapy. Nevertheless, in older standard-risk MB patients, chemotherapy is often not applied after radiotherapy to increase the outcome but to allow further reduction of the irradiation dose to reduce sequelae. Hence, differences in treatment strategies among different categories of patients might influence the outcomes. In addition, a limitation of this study was the use of a dichotomous chemotherapy variable (yes/no). Of course, the combinations of treatments, treatment timing, dose and delivery mode of radiotherapy, and drugs and doses of chemotherapy will influence the patient outcomes. Unfortunately, the number of the various reported drugs and combinations is far too large to conduct subgroup analyses in the present meta-analysis. In addition, some descriptive studies pooled all patients together, irrespective of the exact regimens they performed, and without providing subgroup data.

Of note, several other prognostic factors were reported by various studies, but they could not be summarized in the present meta-analysis. The methylation-derived neutrophil-to-lymphocyte ratio (23), platelet-to-lymphocyte ratio (33), nuclear size (36), fourth ventricular floor involvement (37), ≥20% aggresomes (43), MYC amplification (30, 40), methylation (40), TP53 mutation (40), and loss of chromosome 13 (40) appear to influence OS. Hydrocephalus management (25), fourth ventricular floor involvement (37), ≥20% aggresomes (43), male sex (39), and brainstem involvement (37) might influence EFS. Molecular typing also appears promising for evaluating the prognosis of MB (46, 54–59), but additional studies and meta-analyses will be necessary to determine their prognostic value. Future studies and meta-analyses should examine these factors on the prognosis of MB.

A strength of this meta-analysis is that it included the studies in which the WHO molecular subgrouping has been done in addition to the conventional risk factors. Nevertheless, this meta-analysis also has limitations. First, as for any study that summarizes the results of previous studies, the present meta-analysis inherited the biases and limitations of all included studies. Of note, the various studies used different age cutoffs. In addition, in some hospitals, adolescents are managed by adult services. These factors probably contribute to heterogeneity. Second, there was high heterogeneity in several analyses. Third, some factors were analyzed by a few studies, and the level of evidence was limited. Fourth, the studies were included as long as they presented molecular subtypes. This meta-analysis did not consider the exact methods for subtyping, although it might influence the subtyping results. Finally, RFS could not be analyzed because too few studies examined this survival parameter.

In conclusion, this meta-analysis suggests that the histologic characteristics of MB, molecular subgroups of MB, GTR, and radiotherapy are associated with OS, PFS, and EFS in patients with MB. Nevertheless, the analysis of several parameters was limited by the small number of studies and high heterogeneity. Therefore, high-quality prospective cohort studies are necessary to confirm the results.

## Data Availability Statement

The original contributions presented in the study are included in the article/**Supplementary Material**. Further inquiries can be directed to the corresponding author.

## Author Contributions

Conception and design of study: YL. Acquisition of data: YL and BX. Analysis and/or interpretation of data: YL. Drafting of the manuscript: SL. Revising of the manuscript critically for important intellectual content: JL. Approval of the version of the manuscript to be published: YL, BX, SL, and HL. All authors contributed to the article and approved the submitted version.

## Conflict of Interest

The authors declare that the research was conducted in the absence of any commercial or financial relationships that could be construed as a potential conflict of interest.

## Publisher’s Note

All claims expressed in this article are solely those of the authors and do not necessarily represent those of their affiliated organizations, or those of the publisher, the editors and the reviewers. Any product that may be evaluated in this article, or claim that may be made by its manufacturer, is not guaranteed or endorsed by the publisher.

## Supplementary Material

Supplementary Figure S1 Forest plots of summary prognostic factors and overall survival (OS) **(A)**, progression-free survival (PFS) **(B)**, and event-free survival (EFS) **(C)**.

Supplementary Figure S2 Publication bias. Funnel plot of **(A)** M+ vs. M0, **(B)** sub-total resection (STR) vs. gross-total reduction (GTR) for overall survival (OS).

Supplementary Figure S3 Sensitivity analysis of prognostic factors and overall survival (OS). **(A)** Age. **(B)** Sex. **(C)** Desmoplastic vs. classic. **(D)** Large cell vs. classic. **(E)** M+ vs. M0; **(F)** Sub-total resection (STR) vs. gross-total reduction (GTR). Sensitivity analysis of prognostic factors and progression-free survival (PFS). **(G)** Large cell vs. classic. **(H)** STR vs. GTR. Sensitivity analysis of prognostic factors and event-free survival (EFS). **(I)** M+ vs. M0.
