# Supplementary figures and images for: Risk Factors for Survival in Patients With Medulloblastoma: A Systematic Review and Meta-Analysis
Source: Front Oncol. 2022 Mar 3;12:827054. doi: 10.3389/fonc.2022.827054 (PMC8927734; doi:10.3389/fonc.2022.827054)

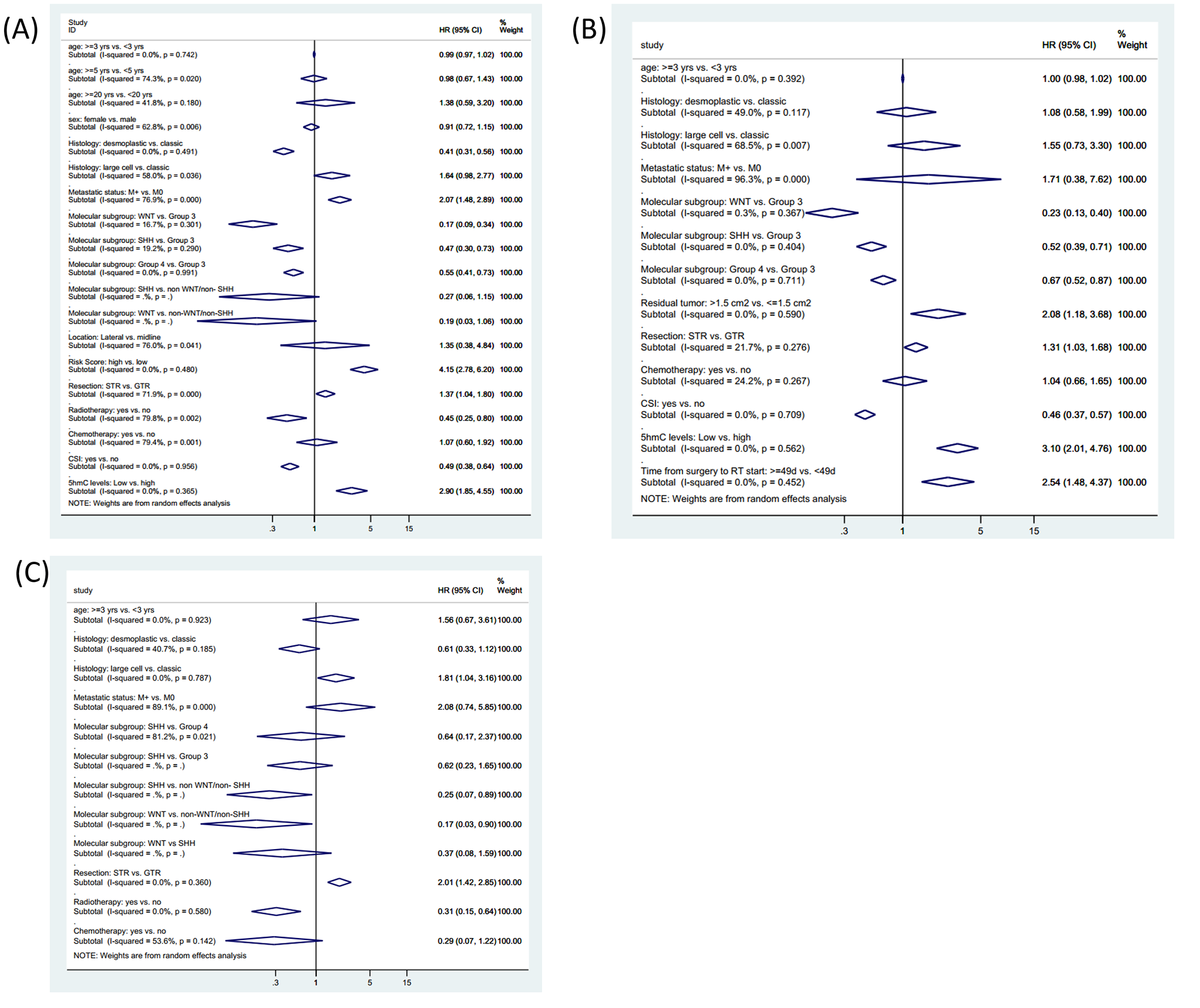

Supplement: Supplementary Figure S1 — Forest plots of summary prognostic factors and overall survival (OS) (A), progression-free survival (PFS) (B), and event-free survival (EFS) (C). [file Image_1.tif]

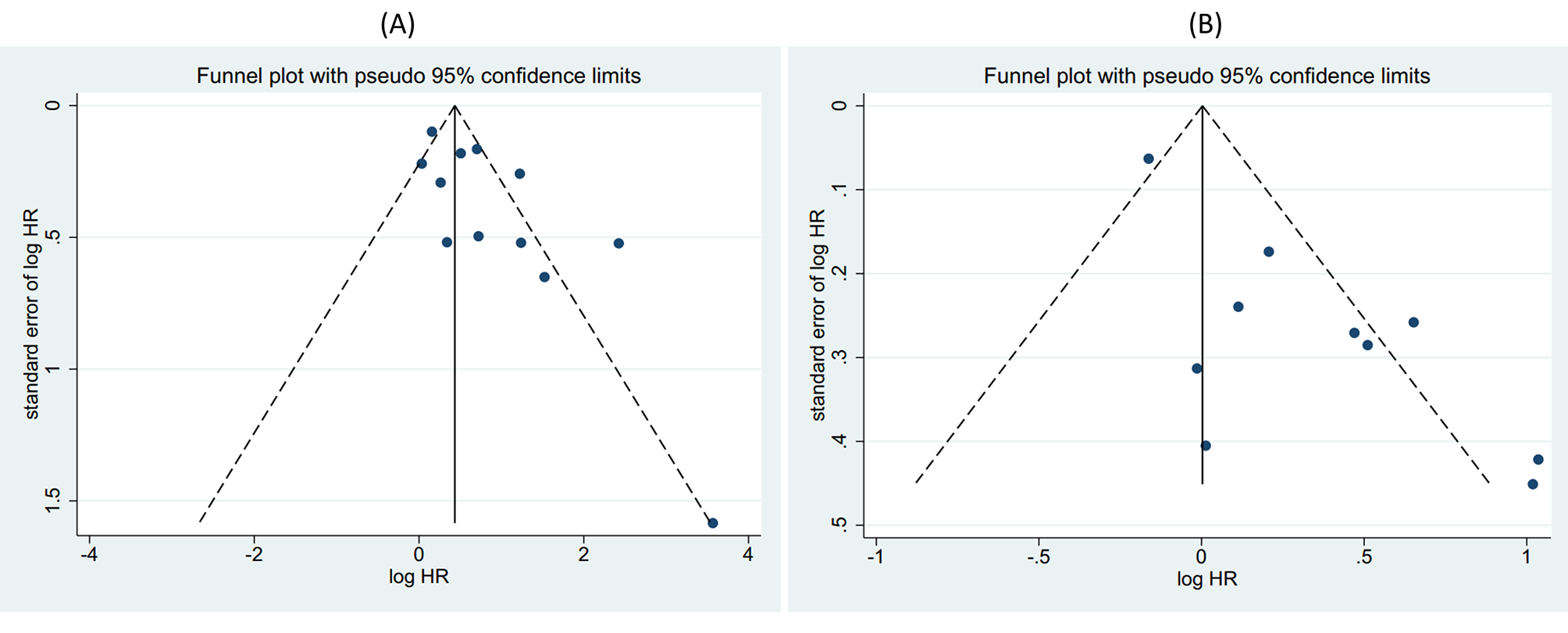

Supplement: Supplementary Figure S2 — Publication bias. Funnel plot of (A) M+ vs. M0, (B) sub-total resection (STR) vs. gross-total reduction (GTR) for overall survival (OS). [file Image_2.tif]

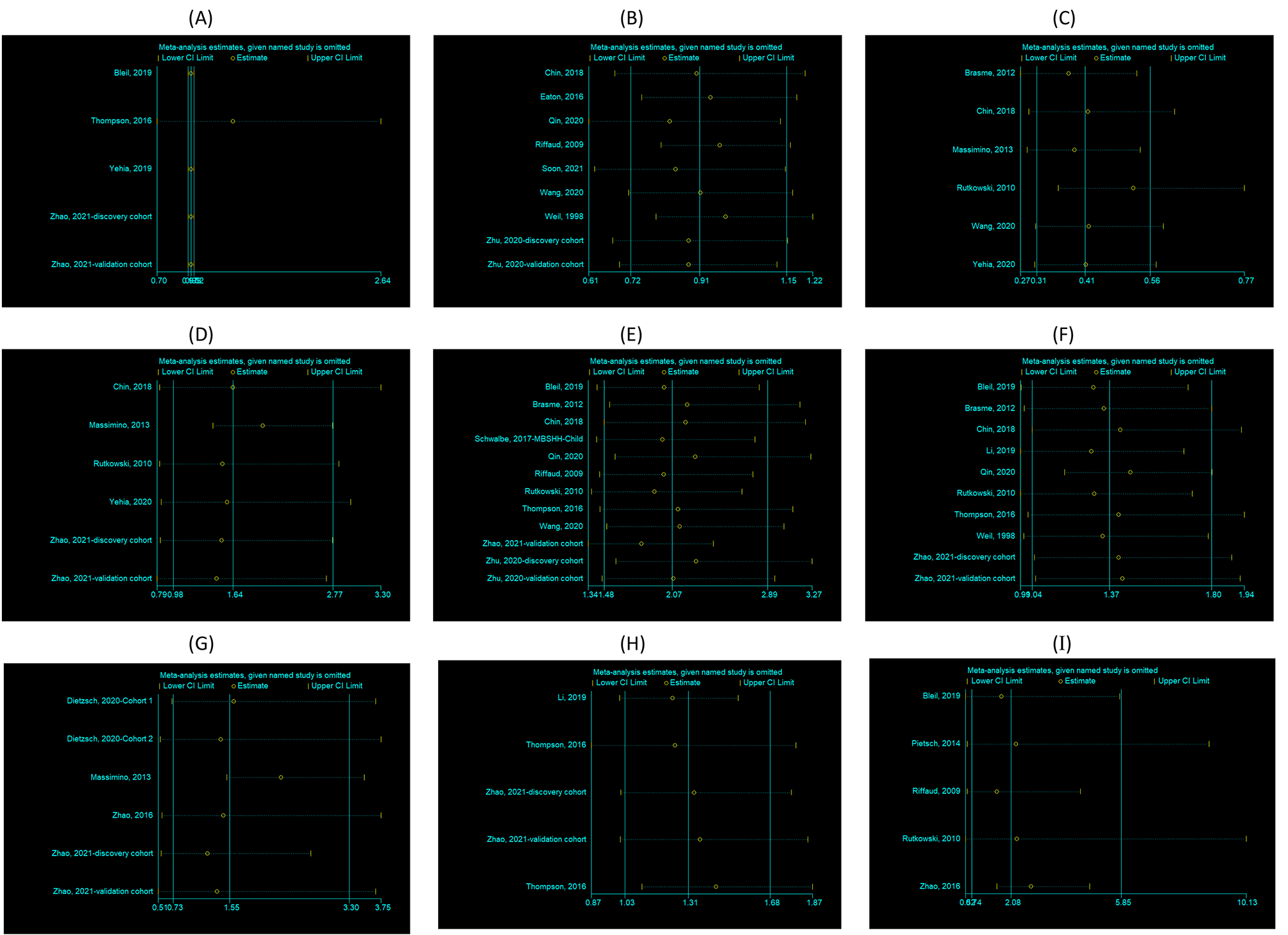

Supplement: Supplementary Figure S3 — Sensitivity analysis of prognostic factors and overall survival (OS). (A) Age. (B) Sex. (C) Desmoplastic vs. classic. (D) Large cell vs. classic. (E) M+ vs. M0; (F) Sub-total resection (STR) vs. gross-total reduction (GTR). Sensitivity analysis of prognostic factors and progression-free survival (PFS). (G) Large cell vs. classic. (H) STR vs. GTR. Sensitivity analysis of prognostic factors and event-free survival (EFS). (I) M+ vs. M0. [file Image_3.tif]
