# Supplementary material for: Risk Factors for Survival in Patients With Medulloblastoma: A Systematic Review and Meta-Analysis
Source: Front Oncol. 2022 Mar 3;12:827054. doi: 10.3389/fonc.2022.827054 (PMC8927734; doi:10.3389/fonc.2022.827054)
Supplement: Supplementary file 4 [file Table_1.doc]

| **Author, year** | **Design** | **Source of subjects** | **Sample size** | **Diagnostic criteria** | **Histology type, n (%)** | **Age at diagnosis (year, mean or median)** | **Adult/Children** | **Location, n (%)** | **Gender, male (%)** | **Extent of resection** | **Radiotherapyn(%)** | **Chemotherapy n(%)** | **Follow up(Year, mean or median)** | **Outcomes** |
| --- | --- | --- | --- | --- | --- | --- | --- | --- | --- | --- | --- | --- | --- | --- |
| Arroyo, 2019 | Cohort | Hospital | 56 | / | / | 8.1/7.1 | Children | / | 64.30% | / | / | 46(82.1) | 6.0 | Survival |
| Back, 2005 | Cohort | Hospital | 182 | Histological | / | 8.1 | Children | Confined to cerebellum132(73) Extension to brain stem 26(14) Extension outside posterior fossa 16(9) Unknown 8(4) | 65.00% | Biopsy only 5(3) STR 75(41) Macroscopic resection 99(54) Unknown 3(2) | 182(100) | 77(42) | 5.3 | RFS |
| Bleil, 2019 | Cohort | Hospital | 69 | Histological | / | 5.83 | Children | Midline (IV ventricle/vermis)44 (81.5) Cerebellar hemisphere 10 (18.5) Presence of hydrocephalus48 (84.2) EVD insertion46 (83.6) Definitive treatment for hydrocephalus 28 (47.5) | / | GTR 43 (76.8) STR 12 (21.4) Biopsy 1 (1.8) Metastatic disease at diagnose 10 (18.2) | 39(68.4) | 51(91.5) | 2.42 | OS, EFS |
| Brasme, 2012 | Cohort | Population | 166 | Histological | standard 78%  DMB 22%  LC/AMB for one patient | ＜5 years 51(30.7) | Children | / | 72.00% | Complete 86 Incomplete 75 No surgery 5 | / | / | 7 | Survival |
| Chin, 2018 | Cohort | Hospital | 1338 | Histological | Classic/NOS 1178(88) DMB 108(8.1) LCMB 52(3.9) | 14–21 years 328(24.5) | Adult/Children | / | 61.30% | GTR 290(21.7) STR or biopsy203(15.2) Unknown 845(63.2) | 1388(100) | 1388(100) | 4.33 | Survival |
| Dietzsch, 2020 | Cohort | Hospital | 382 | Histological | CMB 308 DMB 52 LCMB 22 | ＜5 years 23(6) | Children | / | 62.60% | / | 382(100) | 0 | 9.3 | PFS, OS |
| Eaton, 2016 | Cohort | Hospital | 88 | / | CMB 71 AMB 9 Other 8 | 7.18 | Children | / | 61.40% | / | 88(100) | / | 6.2 for the proton cohort and 7.0 for the photon cohort | OS, RFS |
| Hill, 2020 | Cohort | Hospital | 247 | / | CMB 158(70) DMB 29(13) LCMB 39(17) | ＜4 years 38(15) | Adult/Children | / | 71.00% | / | 42(22) | 206(84) | 4.72 | Time to relapse, OS |
| Lai, 2008 | SEER database | Population | 454 | / | Medulloblastoma 397 (87.44) DNMB 52 (11.45) MB 2 (0.44) LCMB 3 (0.66) | 21–40 341 (75.11) | Adult | / | 59.20% | Biopsy 16 (3.52) STR 91 (20.04) GTR 218 (48.02) Surgery NOS 110 (24.23) Unknown 19 (4.19) | 377 (83.04) | / | / | Survival |
| Li, J., 2020 | Cohort | Hospital | 38 | Immunohistochemistry | / | 8 | Adult/Children | / | 84.00% | / | 9 (24%) | 7 (18%) | / | Survival |
| Li, 2019 | Cohort | Hospital | 116 | Postoperative pathology |  | Children 93 (80.2%) | Adult/Children | Midline 88 (75.9%) Lateral 28 (24.1%) | 64.70% | Gross Total 62 (53.4%) Subtotal 54 (46.6%) | 93 (80.2%) | 64 (55.2%) | / | PFS, OS |
| Massimino, 2013 | Cohort | Hospital | 125 | Histological | / | 8 | Adult/Children | / | 79.20% | / | / | / | 8.17 | PFS, OS |
| Nalita, 2018 | Cohort | Hospital | 55 | Histological | / | 7.05 | Children | Hemisphere 12(21.8) Midline 43(78.2) | 54.50% | Complete resection 17(30.9) | 42(76.4) | 41(74.5) | 5.73 | OS |
| Ozer, 2004 | Cohort | Hospital | 23 | Histological | / | 6 | Children | / | 47.80% | Complete resection 16(69.6) | 23(100) | 23(100) | / | OS |
| Padovani, 2007 | Cohort | Hospital | 248 | Histological | None5 (1) DMB 75 (30) CMB 166 (66) Other7 (3) | 29 | Adult | / | 64.52% | / | 248(100) | 146 (58) | 7 | OS, EFS |
| Pietsch, 2014 | Cohort | Hospital | 184 | / | CMB 132 DMB 37 MBEN 6 LCMB 1 AMB 8 | 7.64 | Adult/Children | / | 65.76% | / | / | / | 1.78 | OS, EFS |
| Qin, 2020 | SEER database | Population | 1589 | Histology code ICD-O3:9470 | / | ＜4, (21.6) | Adult/Children | Brainstem 172 Cerebellar 1417 | 62.90% | No Surgery 34 Biopsy only 2 Surgery, NOS 56 STR 535 GTR 962 | 1271(80) | 1197(75.3) | / | OS |
| Riffaud, 2009 | Cohort | Hospital | 27 | / | CMB 21 DMB 6 | 21 | Adult | Lateral 11 Midline 16 | 40.74% | CR 21 STR or biopsy 6 | / | 7(26) | 8.6 | OS, EFS |
| Rutkowski, 2010 | International Meta-Analysis | Hospital | 260 | Histological | CBM 145 DNMB/MBEN 108 LC/A MB 7 | 1.89 | Children | Midline 198 Hemispheres 59 Unknown 3 | 60.00% | CR 137(52.7) | / | / | 8 | OS, EFS |
| Schwalbe, 2017 | Cohort | Hospital | 230 | / | CMB 154 DN/MBEN 22 LCA 33 MB-NOS 21 | 8.17 | Children | / | 67.80% | GTR169(73.9)  STR 60(26.1) | 32(14) | / | 5.04 | OS |
| Soon, 2021 | SEER database | Population | 1034 | / | / | / | Adult/Children | / | 63.80% | / | / | / | / | OS |
| Thompson, 2016 | Cohort | Hospital | 787 | Histological | / | <3 121(19.3) | Adult/Children | / | 65.20% | GTR 519(65.9) NTR 109(13.9) STR 159(20.2) | 608(77.3) | 688(87.4) | / | PFS, OS |
| Wang, 2020 | Database | / | 62 | / | / | / | / | / | / | / | / | / | / | OS |
| Weil, 1998 | Cohort | Hospital | 109 | Histological | / | 6.14 | Children | / | 57.00% | GTR 59(54.1) STR 39(35.8) Biopsy 11(10.1) | 109(100) | 76(69.7) | / | OS |
| Yehia, 2019 | Cohort | Hospital | 93 | Histological | CMB 50(53.8) D/N 12(12.9) LC/A 31(33.3) | <3 10(10.8) | Children | / | / | GTR 76(81.7) STR 17(18.3) | / | / | / | OS, EFS |
| Yu, 2017 | Cohort | Hospital | 40 | Pathological | / | 5.54 | Children | / | 72.50% | / | / | / | / | OS |
| Zhao, 2016 | Cohort | Hospital | 201 | Histological | CMB 114 DNMB 47 LC/A MB 40 | / | Adult | Midline 112 Hemispheres 89 | 66.17% | GTR 114 STR 87 | / | / | 5 | PFS, OS |
| Zhao, 2021- Discovery cohort | Cohort | Hospital | 81 | Histological | CMB61 (75.3) DNMB 12 (14.8) LC/AMB 8 (9.9) | <3, 12 (14.8) |  | Vermis22 (27.2) Hemisphere 18 (22.2) 4th ventricle 41 (50.6) | 72.80% | GTR 59 (72.8) STR 22 (27.2) | / | 49 (60.5) | / | PFS, OS |
| Zhao, 2021- Validation cohort | Cohort | Hospital | 171 | Histological | CMB 140 (81.9) DNMB 17 (9.9) LC/AMB 14 (8.2) | <3, 16 (9.4) |  | Vermis 59 (34.5) Hemisphere20 (11.7) 4th ventricle 92 (53.8) | 71.90% | GTR 122 (71.3) STR 49 (28.7) | / | 129 (75.4) | / | PFS, OS |

RFS, Relapse-free survival; PFS, Progression-free survival; OS, Overall survival; EFS, Event-free survival; MB, medulloblastoma; LC/A, large cell/anaplastic; DNMB, desmoplastic/nodular MB; DMB desmoplastic MB, LC/AMB, large cell/anaplastic MB; MBEN, non-medulloblastoma with extensive nodularity;MB-NOS, medulloblastoma not otherwise specified ;LCMB, large cell MB; AMB anaplastic MB; D/N, desmoplastic/nodular; CMB, classic MB; GTR, Gross-total resection; NTR, Near-total resection ; STR Sub-total resection; CR, Complete resection.
